# Phylogeographical Studies of *Ascaris* spp. Based on Ribosomal and Mitochondrial DNA Sequences

**DOI:** 10.1371/journal.pntd.0002170

**Published:** 2013-04-11

**Authors:** Serena Cavallero, Viliam Snabel, Francesca Pacella, Vitantonio Perrone, Stefano D'Amelio

**Affiliations:** 1 Department of Public Health and Infectious Diseases, Section of Parasitology, Sapienza University of Rome, Rome, Italy; 2 Institute of Parasitology, Slovak Academy of Sciences, Kosice, Slovakia; 3 Az. USL Roma B, Servizio Veterinario, Dip. di Prevenzione, Rome, Italy; National Institute of Parasitic Diseases China CDC, China

## Abstract

**Background:**

The taxonomic distinctiveness of *Ascaris lumbricoides* and *A. suum*, two of the world's most significant nematodes, still represents a much-debated scientific issue. Previous studies have described two different scenarios in transmission patterns, explained by two hypotheses: (1) separated host-specific transmission cycles in highly endemic regions, (2) a single pool of infection shared by humans and pigs in non-endemic regions. Recently, *A. suum* has been suggested as an important cause of human ascariasis in endemic areas such as China, where cross-infections and hybridization have also been reported. The main aims of the present study were to investigate the molecular epidemiology of human and pig *Ascaris* from non-endemic regions and, with reference to existing data, to infer the phylogenetic and phylogeographic relationships among the samples.

**Methodology:**

151 *Ascaris* worms from pigs and humans were characterized using PCR-RFLP on nuclear ITS rDNA. Representative geographical sub-samples were also analysed by sequencing a portion of the mitochondrial *cox*1 gene, to infer the extent of variability at population level. Sequence data were compared to GenBank sequences from endemic and non-endemic regions.

**Principal Findings:**

No fixed differences between human and pig *Ascaris* were evident, with the exception of the Slovak population, which displays significant genetic differentiation. The RFLP analysis confirmed pig as a source of human infection in non-endemic regions and as a corridor for the promulgation of hybrid genotypes. Epidemiology and host-affiliation seem not to be relevant in shaping molecular variance. Phylogenetic and phylogeographical analyses described a complex scenario, involving multiple hosts, sporadic contact between forms and an ancestral taxon referable to *A. suum.*

**Conclusions/Significance:**

These results suggest the existence of homogenizing gene flow between the two taxa, which appear to be variants of a single polytypic species. This conclusion has implications on the systematics, transmission and control programs relating to ascariasis.

## Author Summary

*Ascaris lumbricoides*, the world's most common human nematode, and *A. suum*, the pig roundworm, are two of the most important soil-transmitted helminthes of public health and socio-economic concern. However, previously documented similarities at the morphological and genetic level, coupled with evidence for hybridization and gene flow, have clouded the taxonomic distinctiveness of these two nematodes. To date, molecular epidemiological studies have been carried out, mostly in highly endemic regions, where two different transmission cycles have been described. Recently, pigs have been recognized as an important source of human ascariasis in China, opening questions about the zoonotic potential and the efficiency of control programs. Here, samples from non-endemic regions have been analysed using a nuclear marker to identify nematodes to species level plus a mitochondrial marker to investigate the phylogeographic relationships among individuals of the two species from both endemic and non-endemic regions. Results obtained suggested that *A. suum* and *A. lumbricoides* may be variants of the same species, with the lack of fixed genetic differences and considerable phylogeographic admixture confirming an extremely close evolutionary relationship among these nematodes. This study highlights the need to further explore the evolutionary affinities of the two taxa to help shed light on the epidemiology of ascariasis.

## Introduction

Ascariasis in pigs and in humans is caused by two of the most socio-economically important nematodes: *Ascaris suum* Goeze, 1782 and *Ascaris lumbricoides* Linneaus, 1758, respectively. Human ascariasis is a soil-transmitted helminthiasis (STH), included in the WHO list of neglected tropical diseases (NTD), infecting more than one billion people [1]. Even if the majority of infections are asymptomatic, clinical manifestations of human ascariasis typically involve acute and chronic symptoms (lung inflammation and fever due to larval migration; abdominal pain, nausea, retarded growth in children and intestinal obstruction due to the massive presence of adult worms) [1]. Ascariasis in pigs is frequent in both intensive and extensive breeding systems, being a source of substantial economic losses [2].

Due to their morphological and biological similarities, the taxonomic distinctiveness of *A. lumbricoides* and *A. suum* still represents a debated scientific issue. Importantly, this issue is of great relevance for both systematists and epidemiologists alike, given its implications on parasite transmission, zoonotic potential, and the establishment of control programs [3], [4], [5]. Several hypotheses have been proposed to explain the origin of the two ascarid taxa in their respective hosts and their taxonomic status [3], namely: a) *A. suum* and *A. lumbricoides* are two valid species; b) *A. suum* is the ancestor of *A. lumbricoides*, originated by an allopatric event of host-switching; c) *A. lumbricoides* is the ancestor of *A. suum*; d) *A. suum* and *A. lumbricoides* are conspecific and therefore occur as variants of a single polytypic species.

Previous molecular epidemiological studies have described two different scenarios in transmission patterns that could be explained by two different hypotheses. First, distinct, host-specific transmission cycles have been observed in highly endemic regions as Guatemala and China [4], [5], [6], [7]. Second, a single pool of infection, shared by humans and pigs, has been observed in non-endemic regions, as Denmark and North America [8], [9]. Conversely, recent results strongly suggest that *A. suum* acts as an important source of human ascariasis in endemic area such as China, where both *Ascaris* spp. co-occur. Here, the authors observed cross-infections and hybridization of human and pig *Ascaris*, thus supporting the second hypothesis on transmission cycles [10].

Considering the uncertain epidemiological picture, the main aim of the present study was to investigate genetic variation in two nuclear and mitochondrial target regions (ITS and *cox*1, respectively) within and among *Ascaris* populations of human and pig origin, collected from a range of non-endemic regions. These molecular data, along with other published sequences available at both local and global scales, were then used to infer the evolutionary, phylogenetic and phylogeographic relationships among samples. The nuclear ribosomal marker (ITS) was chosen to distinguish *A. suum*, *A. lumbricoides* and the hybrid form of the two taxa. Meanwhile, mitochondrial DNA is the most frequently used molecular marker in this kind of studies, due to desirable biological features such as maternal inheritance, high mutation rate, very low recombination rate, haploidy, and putative selective neutrality, making mtDNA markers particularly suitable as barcoding tools to identify sibling and cryptic species [11], [12].

Studies aimed at investigating the molecular epidemiology of ascariasis are important not only to clarify the transmission patterns of the two roundworms, but also to better quantify the level of gene introgression between host-associated populations [10]. Such knowledge is important, given that introgression often results in the selection of novel genes, the promotion of rapid adaptive diversification, and homogenization across the genomes of the interbreeding populations [13], [14]. Additional sources of information are now available from the recently published draft genome of *A. suum* [15].

## Methods

### Samples

A total of 151 adult nematodes belonging to *Ascaris* spp. were collected from pig (n = 143) and human (n = 8) hosts. Nematodes collected were repeatedly washed in saline and stored in 70% ethanol. Collection data including collecting sites, hosts, number of parasites specimens analysed and identification codes are summarised in Table 1.

**Table 1:** Details of specimens analysed by PCR-RFLP approach on ITS ribosomal nuclear amplicons.

Geographical Origin	Host	Np	Codes	Ng	As	Al	H
Italy	Pig	70	ASI1-11_S	60	49	2	9
			ASI14-31_S				
			ASI33-73_S				
	Human	3	ASI12_U	2	1		1
			ASI13_U				
			ASI32_U				
Slovakia	Pig	45	ASS1-45_S	44	36	4	4
Hungary	Pig	28	ASU1-28_S	27	19		8
Syria	Human	1	AUS_U	1		1	
Romania	Human	2	ASR1-2_U	2	1	1	
Pakistan	Human	2	ASP1-2_U	2		2	
Total		151		137	106	10	22
					(77%)	(7%)	(16%)

Geographical origin, host, number of parasites collected (Np), codes, number of parasites successfully genotyped (Ng), number of “*suum*” genotype (As), number of “*lumbricoides*” genotype (Al) and number of “heterozygote” genotype (H).

DNA was isolated using the Wizard Genomic DNA purification kit (Promega) according to the manufacturer's protocol.

### Ethical statement

All samples, from human and animal origin, were obtained from existing collections. Samples from human origin were obtained from existing collections at Tor Vergata and Sant'Andrea Polyclinics in Rome. Data collection includes only the geographical origin of patients and no reference to personal data was recorded, thus guaranteeing the absolute anonymity of these specimens.

Sample collection at the Polyclinics that provided the nematodes from humans was performed in concordance with the WMA Helsinki Declaration (Edinburgh 2000) and its subsequent modification, as well as with the Italian National Law n. 675/1996 on the protection of personal data.

### PCR-RFLP of ITS region

The entire ITS nuclear region (ITS1, 5.8S, ITS2) was amplified using 5.0 µl of template DNA (20–40 ng), 10 mM Tris-HCl (pH 8.3), 1.5 mM MgCl_2_ (Bioline), 40 mM of a nucleotide mix (Bioline), 50 pmol/µl each of the forward primer NC5 (5′-GTAGGTGAACCTGCGGAAGGATCAT-3′) and the reverse primer NC2 (5′-TTAGTTTCTTCCTCCGCT-3′) described by Zhu et al.[16] and 1.0 U of BIOTAQ DNA Polymerase (Bioline) in a final volume of 50 µl. The PCR was performed in a GenePro Eurocycler Dual Block (Bioer) under the following conditions: 10 min at 95°C (initial denaturation), 30 cycles of 30 sec at 95°C (denaturation), 40 sec at 52°C (annealing) and 75 sec at 72°C (extension), and a final elongation step of 7 min at 72°C. A negative control (without genomic DNA) was included in each set of amplification reactions.

A representative subset of specimens (Table 2) was also analysed by sequencing a portion of the mitochondrial cytochrome oxidase I gene (*cox*1), after amplification using the forward primer As-Co1F (5′-TTTTTTGGTCATCCTGAGGTTTAT- 3′) and the reverse primer As-Co1R (5′-ACATAATGAAAATGACTAACAAC- 3′), as described by Peng et al. [6], under the following conditions: 5 min at 94°C, followed by 35 cycles of 94°C for 30 s; 45 s at 55°C; 90 s at 72°C, followed by 5 min at 72°C. Aliquots (5 µl) of individual PCR products were separated by electrophoresis using agarose gels (1%), stained with ethidium bromide (0.4 µg/ml) and detected using ultraviolet trans-illumination.

**Table 2:** Details of haplotypes recovered in the partial *cox*1 sequence analyses.

Haplotype	Cluster	GenBank	Codes	ITSg	Host	Region	Haplotype frequency		
							Slovakia	Italy	Hungary
Hap1	C	KC455923	ASS1,2,4,5,9,15,19	As	Pig	NE	0.429	0.0769	
	C		ASS6		Pig	NE			
	C		ASI11	H	Pig	NE			
	C		ASI14	As	Pig	NE			
	C	JN575632	ASS47		Pig	NE			
Hap2	C	KC455924	ASS3, 8	As	Pig	NE	0.0952		
Hap3	C	KC455925	ASS7,11,12,18	As	Pig	NE	0.333		
	C		ASS10	Al	Pig	NE			
	C		ASS16	H	Pig	NE			
	C	JN575633	ASS46		Pig	NE			
Hap4	C	KC455926	ASS14	As	Pig	NE	0.0476		
Hap5	A1	KC455927	ASS20	As	Pig	NE	0.0476	0.615	0.875
	A1		ASU2,4,6,7,10,11,20,21	As	Pig	NE			
	A1		ASU5		Pig	NE			
	A1		ASU8,9,12,27,28	H	Pig	NE			
	A1		ASI3,4,10,15–17	As	Pig	NE			
	A1		ASI12	As	Human	NE			
	A1		ASI18–26		Pig	NE			
	A1		ASR1	As	Human	NE			
	A1	AJ968334	ASC18		Pig	E			
Hap6	A1	KC455928	ASU1	As	Pig	NE			0.0625
Hap7	B	KC455929	ASI8,9	As	Pig	NE		0.0769	
	B	JN575631	ASS48		Human	NE			
	B	GU326952	ASB3		Human	NE			
	B	AB591804	ASG1		Pig	E			
	B	AB591802	ASG2		Pig	NE			
	B	AB591805	ASG6		Pig	NE			
	B	AB591800	ASG3		Human	NE			
	B	AB591798	ASG4		Human	NE			
	B	AB591796	ASG5		Human	NE			
	B	AB591799	ASG9		Human	NE			
	B	AB591797	ASG10		Human	NE			
	B	EU582490	ASZ5		Human	E			
	B	AJ968342	ASC11		Pig	E			
	B	AJ968338	ASC13		Pig	E			
	B	AJ968332	ASC14		Human	E			
Hap8	A1	KC455930	ASU3	As	Pig	NE			0.0625
Hap9	A1	KC455931	ASI1	As	Pig	NE		0.0385	
Hap10	A1	KC455932	ASI2	As	Pig	NE		0.0385	
Hap11	A1	KC455933	ASI5	As	Pig	NE		0.115	
	A1		ASI6,7	H	Pig	NE			
Hap12	A2	KC455934	ASI13		Human	NE		0.0385	
	A2	GU326951	ASB1		Pig	E			
	A2	GU326949	ASB4		Human	E			
	A2	GU326948	ASB7		Human	E			
	A2	EU582492	ASZ4		Human	E			
	A2	EU582484	ASZ8		Human	E			
	A2	EU582497	ASZ10		Human	E			
	A2	AJ968336	ASC17		Pig	E			
Hap13	A2	GU326954	ASB2		Human	E			
Hap14	B	GU326953	ASB5		Human	E			
Hap15	A	GU326955	ASB6		Human	E			
Hap16	A2	HM602025	ASB8		Pig	E			
Hap17	A	AB591803	ASG7		Pig	NE			
Hap18	A2	AB591801	ASG8		Human	NE			
Hap19	B	AB591795	ASG11		Human	NE			
Hap20	A2	EU582498	ASZ1		Human	E			
Hap21	A2	EU582496	ASZ2		Human	E			
Hap22	A2	EU582494	ASZ3		Human	E			
Hap23	A2	EU582488	ASZ6		Human	E			
Hap24	B	EU582486	ASZ7		Human	E			
Hap25	B	EU582499	ASZ9		Human	E			
Hap26	B	EU582493	ASZ11		Human	E			
	B	AJ968340	ASC12		Pig	E			
Hap27	A2	EU582495	ASZ12		Human	E			
Hap28	A1	EU582491	ASZ13		Human	E			
Hap29	A2	EU582489	ASZ14		Human	E			
Hap30	A2	EU582487	ASZ15		Human	E			
Hap31	A2	EU582485	ASZ16		Human	E			
Hap32	B	AJ968343	ASC1		Pig	E			
Hap33	B	AJ968341	ASC2		Pig	E			
Hap34	B	AJ968339	ASC3		Pig	E			
Hap35	B	AJ968337	ASC4		Pig	E			
Hap36	A1	AJ968335	ASC5		Pig	E			
Hap37	A1	AJ968333	ASC6		Human	E			
Hap38	B	AJ968331	ASC7		Human	E			
Hap39	A1	AJ968329	ASC8		Human	E			
Hap40	A	AJ968327	ASC9		Human	E			
Hap41	A2	AJ968325	ASC10		Human	E			
Hap42	B	AJ968330	ASC15		Human	E			
Hap43	A1	AJ968328	ASC16		Human	E			
Hap44	A2	AJ968326	ASC19		Human	E			
Hap45	A1	AJ968324	ASC20		Human	E			
Hap46	B	KC455935	ASP	Al	Human	E			

Haplotypes, affiliation to phylogenetic clusters A(A1, A2)-B-C, GenBank accession numbers, codes, genotypes recovered using RFLP approach on ITS ribosomal nuclear amplicons (“*suum*” genotype: As, “*lumbricoides*” genotype: Al and “heterozygote” genotype: H), host, region (NE: non-endemic; E: endemic). Haplotypes relative frequencies are reported only for populations of Dataset1.

Positive ITS amplicons were digested with the restriction endonuclease *Hae*III, as the resulting patterns have been previously proved useful for the identification of human and pig *Ascaris* species [8]. Digests were resolved by electrophoresis in 2% agarose gels, stained with ethidium bromide (0.4 µg/ml), detected under UV trans-illumination, and the fragments sizes determined by comparison with a 100 bp DNA ladder (Promega). Information on geographical origin, hosts, codes, number of parasites successfully genotyped, and genotypes recovered using PCR-RFLP are available in Table 1.

### Phylogenetic and network analysis of *cox*1 region

Positive amplicons were purified by SureClean (Bioline), following the manufacturer's instructions, and then sequenced by MWG Eurofins DNA. Two different datasets were created, each representing different partial *cox*1 alignments: the first including only samples analysed in the present paper (Dataset1), with the exclusion of two human nematodes due to small sample size (single specimens from Pakistan and Romanian human patients), and the second including all GenBank retrieved sequences of specimens collected from endemic and non-endemic regions (Dataset2). Information about specimens sequenced for *cox*1, identification codes and accession numbers, also of GenBank retrieved sequences are available in Table 2.

Nucleotide sequences were aligned using Clustal X implemented in MEGA 5 [17] and then analysed using DnaSP v5 [18] to infer haplotype composition. In addition, sequences were analysed using Arlequin 3.11 [19] to estimate several variability indexes: the relative frequencies of haplotypes; population differentiation (F_ST_) among samples for Dataset1; hierarchical analyses of molecular variance (AMOVA) to evaluate the amount of population genetic structure for Dataset2, using information on the allelic content of haplotypes, as well as their frequencies. The significance of the covariance components associated with the different levels of genetic structure (within individuals of populations, among populations and among groups) was tested using non-parametric permutation procedures [20]. The AMOVA was undertaken twice, using two different criteria to define groups and population structure: geographical origin (endemic and non-endemic regions) and host affiliation (pig and human).

Both Dataset1 and 2 were also analysed using a phylogenetic approach based on Bayesian reconstruction method. The program JModeltest [21] was used to compare the fit of nucleotide substitution models using the Akaike Information Criterion (AIC), under a total of 83 models, corresponding to 11 different schemes; the best-fit model and parameters determined for both *cox1* datasets were then used for the Bayesian analyses. The Bayesian analyses were performed using the HKY+I model for both datasets (as selected by ModelTest), using BEAST software [22]; datasets were run twice for 10^6^ generations. Posterior probability values (BPP) shown in the Bayesian consensus trees were determined after discarding trees from the burn-in period. For each dataset, burn-in was estimated to include the first 10^4^ generations. A second phylogenetic method was performed only on Dataset 2 using MEGA5 [23]: the evolutionary distances were computed using the Tamura-Nei [24] with Neighbor joining method (NJ) and statistical support at nodes was evaluated using 1000 pseudoreplication bootstrap [25]. Phylogenetic trees included *Anisakis* Dujardin 1845 as outgroup (GenBank accession number: JN102304).

Moreover, statistic parsimony networks [26] using TCS software [27] were inferred for both datasets in order to determine the phylogeographic distribution and genealogy of the *Ascaris* specimens analysed, running the network at a 95% connection limit, which is the maximum number of mutational connections between pairs of sequences justified by the parsimony criterion.

## Results

### PCR-RFLP ITS analysis

**Figure 1:**
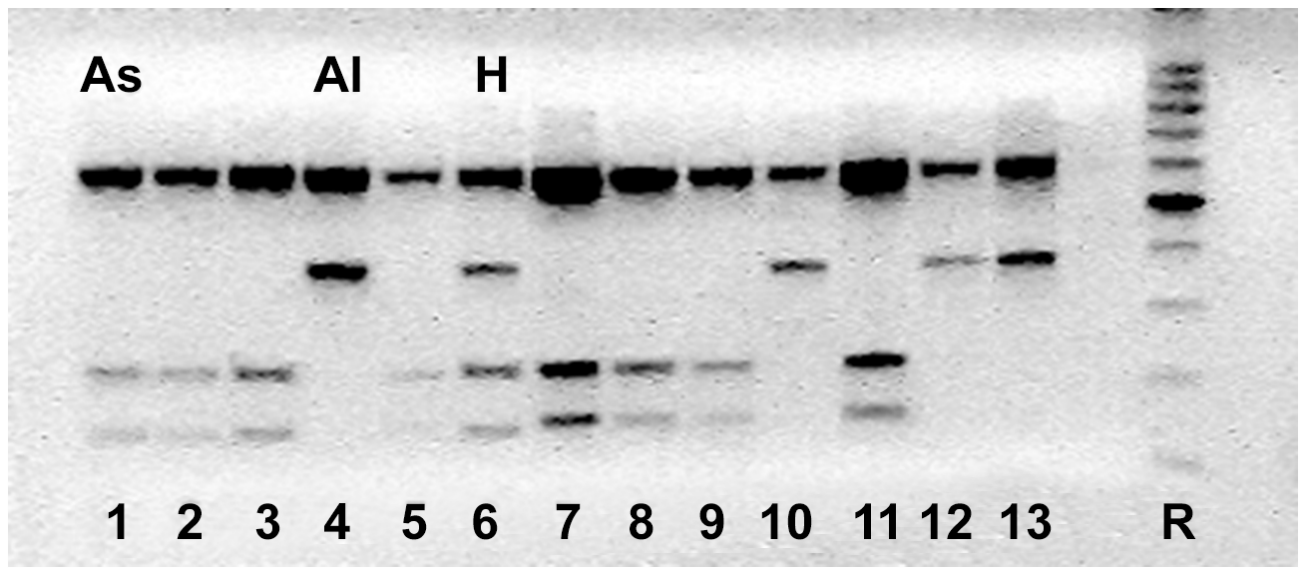
Molecular characterization of *Ascaris* by PCR-RFLP. A representative gel displaying the RFLP profiles following the digestion of ITS amplicons with restriction endonuclease *Hae*III. Genotype As: *Ascaris suum* banding pattern (lanes 1–3,5,7–9,11); genotype Al : *A. lumbricoides* banding pattern (lanes 4,10,12,13); genotype H: hybrid/heterozygote banding pattern (lane 6); R (right-hand lane): 100bp reference ladder.

A PCR product of around 1000 bp was obtained for 137 of the 151 specimens analysed. Amplicons were subsequently digested using the *Hae*III restriction enzyme. This approach yielded the identification of three genetically distinct banding patterns belonging to the genus *Ascaris*: the “*lumbricoides”* genotype displays two bands of about 610 bp and 370 bp, the “*suum*” genotype shows three bands of about 610 bp, 230 bp and 140 bp, and the “hybrid” genotype displays all the four bands mentioned above (Figure 1).

While the proportion of each genotype varied somewhat across the various localities sampled, all regions revealed instances of discordance between the expected genotype and host of origin (Table 1). For Italy, although 49 of 60 positive samples from pigs displayed the expected “*suum*” genotype, nine displayed the “hybrid” genotype and two displayed the “*lumbricoides*” genotype. In contrast, neither of the two positive human isolates displayed the expected “*lumbricoides*” pattern, instead revealing one “*suum*” and one “hybrid” genotype. Positive samples obtained from nematodes collected in other countries included four specimens from humans and 71 from pigs. Of the human nematodes, three specimens (Syrian, Pakistan and Romanian patients) showed the typical “*lumbricoides*” genotype and one (another Romanian patient) displayed the “*suum*” genotype. Among Slovak pigs (n = 44), 36 showed the “*suum*” genotype, four the “*lumbricoides*” genotype, and four the “hybrid” pattern, while Hungarian pigs (n = 27) included 19 specimens and eight specimens displaying the “*suum*” genotype and “hybrid” genotypes, respectively. Overall, the “hybrid” genotype was encountered in specimens from both pig and human hosts, at a frequency of 16%.

### *Cox*1 phylogenetic and network analyses

A PCR product of around 400 bp was obtained for 62 specimens amplified. The alignments of Dataset1 (62 sequences) and Dataset2 (120 sequences) yielded a usable alignment of 327 bp. Representative sequences for each haplotype recovered in the course of the present study are available in GenBank under the following Accession Numbers: Hap1: KC455923, Hap2: KC455924, Hap3: KC455925, Hap4: KC455926, Hap5: KC455927, Hap6: KC455928, Hap7: KC455929, Hap8: KC455930, Hap9: KC455931, Hap10: KC455932, Hap11: KC455933, Hap12: KC455934, Hap46: KC455935.

Twelve haplotypes were identified in Dataset1 (Hap1-12), with a total haplotype diversity (Hd) of 0.70 (haplotypes recovered were deposited in GenBank, see Table 2 for accession numbers). Five haplotypes were observed in Slovak sample, with Hd = 0.71; three haplotypes were observed in Hungarian sample, with Hd = 0.24 and seven haplotypes were observed in Italian sample, with Hd = 0.62. The most frequent haplotype was Hap5, shared among the Italian (frequency of 61.5%), Hungarian (87.5%) and Slovak samples (5.5%). Hap1 was the most frequent haplotype in the Slovak population (44.4%) and it has been less frequently reported also in Italian specimens (7.7%). Results from F_ST_ analysis showed significant differences between Slovak sample and the Italian (0.29) and Hungarian samples (0.49), and little differentiation between Italian and Hungarian samples (0.05). Considering Dataset2, forty-five haplotypes were identified, with Hd = 0.89; Hap5 was observed also in the Chinese pig sample. The Italian and Slovak samples showed haplotype Hap7 in common with endemic (Brazil, Zanzibar and China) and non-endemic regions (Japan); the Italian sample showed also haplotype Hap12 in common with endemic regions. Information about haplotypes recovered in the partial *cox*1 sequences analyses, haplotype affiliation to phylogenetic clusters A(A1, A2)-B-C, GenBank accession numbers, codes, correspondences to genotypes identified using RFLP approach on ITS, hosts, endemic and non-endemic origin of samples and haplotypes relative frequencies for populations of Dataset1 are available in Table 2.

AMOVA analysis suggested a higher influence of the epidemiological (endemic/non-endemic origin) criterion in modulating the accumulation of variability with respect to host affiliation, even if the percentage of variation at group level was not significant (3.83% and 0.10%; p = 0.38 and 0.61, respectively). Significant values (p≤0.05) were obtained for the variation observed among populations within groups and among individuals within populations in both analyses, but with an opposite trend: percentage of variation within population was higher than among populations of the same group if the endemic/non-endemic criterion is considered as feature to group samples.

**Figure 2:**
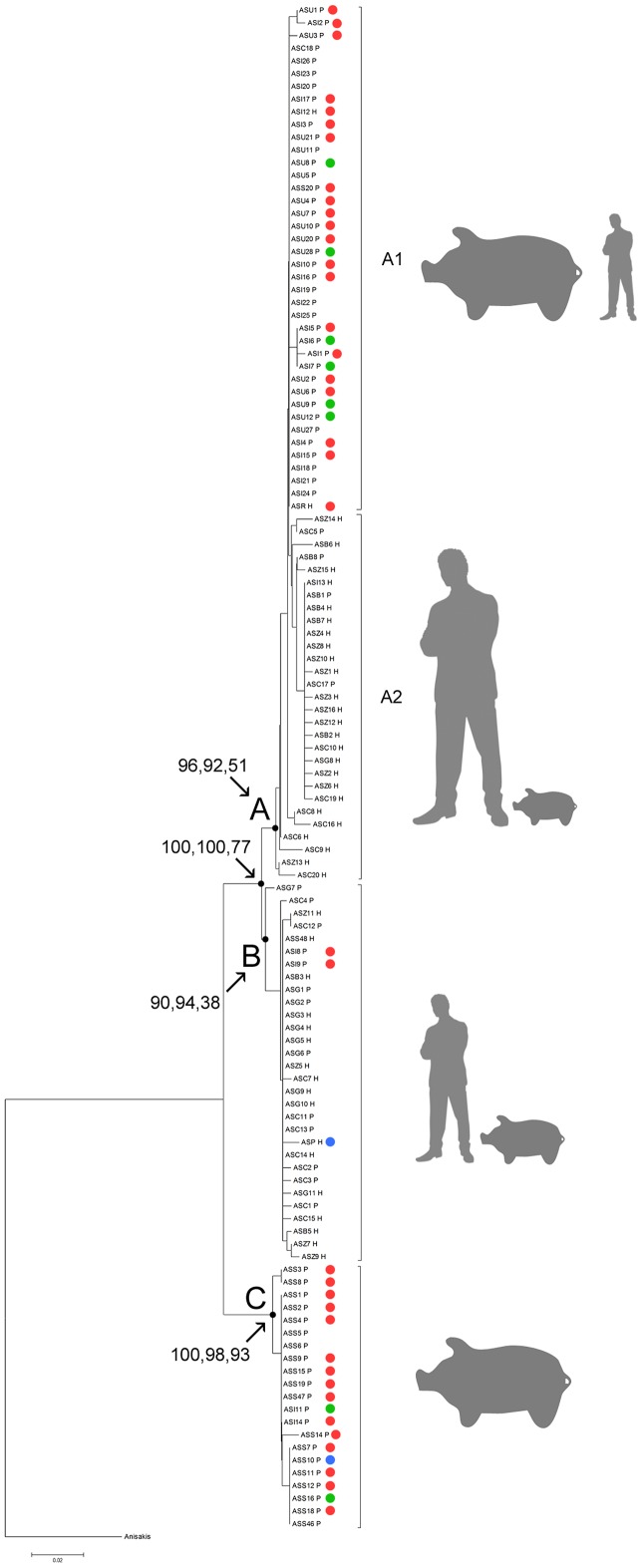
Neighbor Joining tree of *Ascaris* samples. Evolutionary history of the *Ascaris* sp. partial *cox1* mitochondrial DNA sequences, obtained by Neighbor Joining analysis. The same topology was obtained for Bayesian analyses. Numbers at nodes branches represent posterior probability (BPP) values of Dataset 2, Dataset 1 and bootstrap values (A = Cluster A, including subclusters A1 and A2; B = Cluster B; C = Cluster C). The bar shows the mean number of base substitution per site. H and P in codes represent human and pig hosts, respectively. Colored dots indicate ITS RFLP genotypes (blue: *A. lumbricoides*, red: *A. suum*, green: hybrid genotype). Trend for host affiliation is indicated on the right of the tree, as defined by the proportional size of human and pig gray figures.

Bayesian and NJ phylogenetic analyses, based on Dataset1 and Dataset2, described similar topologies, with three main clusters (Figure 2), analogous to the clusters named A, B and C in Anderson and Jaenike [28] and Snabel et al. [29] studies. Clusters A and B have been recently reported also by Iniguez et al. [30].

Cluster A includes samples from both pigs and humans collected from endemic and non-endemic zones; it showed further slight internal subdivision according to host affiliation and epidemiological features, although no statistical support for this partitioning was found. Sub-cluster A1 contains mainly specimens from pigs and few from humans, collected from non-endemic zones. It is important to underline that the specimens of human origin (ASR_H and ASI12_H) included in this group showed the typical “*suum*” genotype for PCR-RFLP analysis of the ITS region. Sub-cluster A2 includes mainly specimens from humans collected from endemic areas, except for one human sample (ASI13 corresponding to Hap12) collected from non-endemic regions, although the country origin of the patient is unknown. Cluster B is also characterized by the presence of specimens from both pigs and humans collected from endemic (Brazil, China, Zanzibar, Pakistan) and non-endemic zones (Japan, Italy). Cluster C comprises only specimens from pig collected from non-endemic regions (Italy and Slovakia). It appears to be well separated from clusters A and B that are more closely related to each other. The existence of the three clusters is well supported by very high posterior probability values (BPP ranging from 92 to 98 for Dataset1 and from 90 to 100 for Dataset2); NJ tree bootstrap values show high statistical support for cluster C (93) and lower values for cluster A (51) and B (38), nevertheless the value supporting the distinctiveness of cluster C from A and B together is fairly high (77).

**Figure 3:**
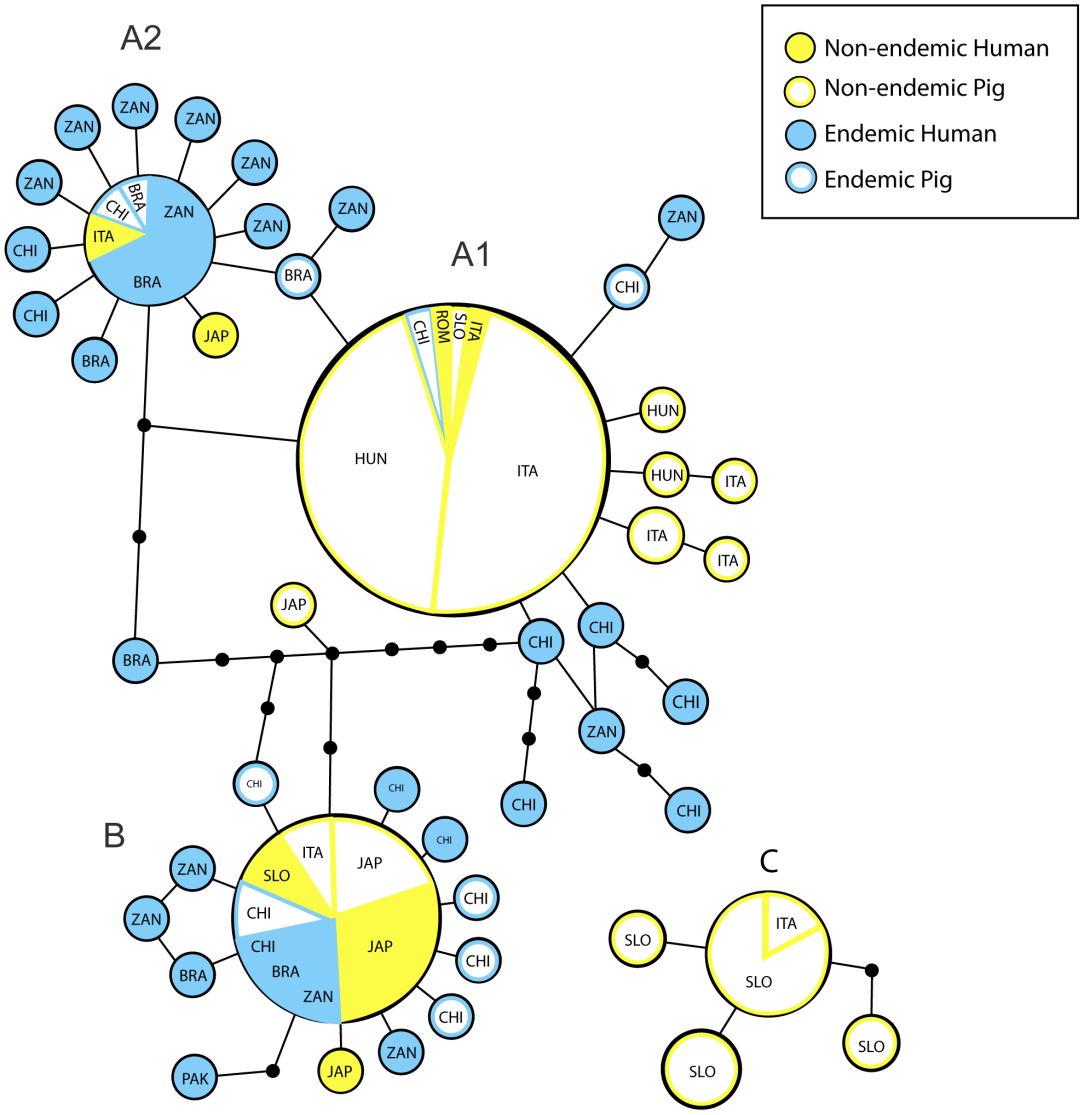
Parsimony network of *Ascaris* haplotypes. Parsimony network of the haplotypes observed in the Dataset2, with 95% connection limit. Circles diameter is proportional to haplotypes frequency; circles at branches represent SNPs (A = Cluster A, including subclusters A1 and A2; B = Cluster B; C = Cluster C).

Results obtained from parsimony network analysis on Dataset2 (Figure 3) describes a very complex scenario where the three clusters observed in phylogenetic analysis are recognized and the slight subdivision inside cluster A is still evident. The main haplogroup, where Hap5 is the more frequent and typically associated to *A. suum*, corresponds to cluster A1 with several haplotypes branching around. The star-like distribution of haplotypes is also evident in the other haplogroups, represented by Hap12 for cluster A2 and Hap7 for cluster A. Cluster A2 is mainly represented by haplotypes from endemic regions, typically associated to *A. lumbricoides*, with the exception of Italian and Japanese human cases; while cluster B includes both pig and human specimens from endemic and non-endemic regions. The Slovak haplogroup appears completely separated from the other haplotypes. These results confirm the relationships observed in the Bayesian phylogenetic trees.

## Discussion

Human and pig *Ascaris* spp. are two of the world's most common soil-transmitted parasites and together cause serious health and socio-economic problems. Ascariasis is considered a NTD as it occurs commonly in rural and poor urban areas and promotes poverty due to its high impact on child health and development, pregnancy and worker productivity. Similarities in the morphology and biology of these two nematodes entail ongoing ambiguity concerning their taxonomic status and argue for the need to delve deeper into their comparative molecular epidemiology.

The present paper provides additional information on the molecular epidemiology of ascariasis in non-endemic regions, such as Italy and Eastern Europe. Molecular characterization using a PCR-RFLP approach on a nuclear marker has confirmed that most pig nematodes sampled herein displayed the typical *A. suum* pattern, corresponding to the genotype G3, while the two human nematodes from endemic regions such as Pakistan and Syria showed the typical *A*. *lumbricoides* pattern, corresponding to the genotype G1 [31]. Cross-infection is confirmed in both hosts by instances of *A. suum* genotypes in human nematodes and *A*. *lumbricoides* in pigs. Moreover, a significant percentage of nematodes displaying the “hybrid” pattern, corresponding to the G2 genotype [31], has been observed in both human and pig nematodes, strongly inferring the presence of gene flow between the two taxa. This combined evidence suggests that *Ascaris suum* can function as a relevant agent of human infection in non-endemic areas. These data are in agreement with recent results described firstly by Betson et al. in Zanzibar [32] and then by Zhou et al. in China [10], where zoonotic transmission of *A. suum* is suggested to occur also in these endemic areas. The zoonotic potential of *A. suum* therefore needs to be reevaluated in order to plan more efficient control programs.

Phylogenetic analyses revealed the homology to the clusters previously observed in Anderson and Jaenike [28] and in Snabel et al. [29], confirming that geographical origin plays an important role in structure of cluster A, where endemic and non-endemic samples split in two sub-clades, but not in cluster B, which contains specimens from both epidemiologically classified regions. Finally, significant values on population differentiation analysis and high haplotype diversity confirm the genuine separation of cluster C. As these parameters are important indexes for evaluating genetic diversity and differentiation, further analysis will be required to understand the significance of this pronounced genetic dissimilarity.

Phylogeographic analyses are helpful in understanding population differentiation, species formation and ecological adaptation [33]. Results obtained from the haplotype network analysis have revealed a very complex scenario: the typical *A. suum* haplotype is the most frequent among samples from non-endemic regions plus is observed also in human patients (circle A1); moreover, this haplogroup is closely related to the haplogroup including the distinctive *A. lumbricoides* haplotypes found in endemic regions (circle A2), which is related in turn to a mixed group homologous to cluster B obtained in phylogenetic inferences (circle B). The picture described a cross-linked relationships among haplotypes, where no clear geographical or host-affiliation criteria seem to be relevant in shaping haplogroups. Shared haplotypes between pig and human *Ascaris* spp. could be explained by evolutionary processes such as introgression and/or retention of ancestral polymorphisms, as suggested previously [9], [34]. In addition, molecular variance analysis underlined that accumulation of genetic variability is observed at the individual and population level rather than at the level of groups defined on geography or host-affiliation.

The overall results showed no fixed differences between human and pig *Ascaris*, describing two taxonomic entities intimately interconnected and therefore likely to experience gene flow. These data strongly infer the absence of a major genetic barrier between the two taxa and therefore suggest that *A. suum* and *A. lumbricoides* may be variants of the same species, as suggested by Leles et al. [3] and Liu et al. [35], and more recently by Iniguez et al. [30]. Together all four studies have found no evidence of diagnostic genetic heterogeneity between human and pig *Ascaris*, plus an absence of genetic clusters discriminating each host.

## Supporting Information

Table S1 **Haplotype relative frequencies.**
(DOC)
