# Supplementary material for: Phylogeographical Studies of Ascaris spp. Based on Ribosomal and Mitochondrial DNA Sequences
Source: PLoS Negl Trop Dis. 2013 Apr 11;7(4):e2170. doi: 10.1371/journal.pntd.0002170 (PMC3623706; doi:10.1371/journal.pntd.0002170)
Supplement: Table S1 — Haplotype relative frequencies. (DOC) [file pntd.0002170.s001.doc]

**Supplementary material 1**:

Table S1: Haplotype relative frequencies observed in Dataset 2. n: number of individuals for each populations.

Haplotype Slovak Hungary Italy Brasil japan zanzibar china

n (21) (16) (26) (8) (11) (16) (20)

-----------------------------------------------------------------------------------------------------------

Hap_1 0.429 0 0.0769 0 0 0 0

Hap_2 0.0952 0 0 0 0 0 0

Hap_3 0.333 0 0 0 0 0 0

Hap_4 0.0476 0 0 0 0 0 0

Hap_5 0.0476 0.875 0.615 0 0 0 0.05

Hap_6 0.0476 0 0.0769 0.125 0.727 0.0625 0.15

Hap_7 0 0.0625 0 0 0 0 0

Hap_8 0 0.0625 0 0 0 0 0

Hap_9 0 0 0.0385 0 0 0 0

Hap_10 0 0 0.0385 0 0 0 0

Hap_11 0 0 0.115 0 0 0 0

Hap_12 0 0 0.0385 0.375 0 0.188 0.05

Hap_13 0 0 0 0.125 0 0 0

Hap_14 0 0 0 0.125 0 0 0

Hap_15 0 0 0 0.125 0 0 0

Hap_16 0 0 0 0.125 0 0 0

Hap_17 0 0 0 0 0.0909 0 0

Hap_18 0 0 0 0 0.0909 0 0

Hap_19 0 0 0 0 0.0909 0 0

Hap_20 0 0 0 0 0 0.0625 0

Hap_21 0 0 0 0 0 0.0625 0

Hap_22 0 0 0 0 0 0.0625 0

Hap_23 0 0 0 0 0 0.0625 0

Hap_24 0 0 0 0 0 0.0625 0

Hap_25 0 0 0 0 0 0.0625 0

Hap_26 0 0 0 0 0 0.0625 0.05

Hap_27 0 0 0 0 0 0.0625 0

Hap_28 0 0 0 0 0 0.0625 0

Hap_29 0 0 0 0 0 0.0625 0

Hap_30 0 0 0 0 0 0.0625 0

Hap_31 0 0 0 0 0 0.0625 0

Hap_32 0 0 0 0 0 0 0.05

Hap_33 0 0 0 0 0 0 0.05

Hap_34 0 0 0 0 0 0 0.05

Hap_35 0 0 0 0 0 0 0.05

Hap_36 0 0 0 0 0 0 0.05

Hap_37 0 0 0 0 0 0 0.05

Hap_38 0 0 0 0 0 0 0.05

Hap_39 0 0 0 0 0 0 0.05

Hap_40 0 0 0 0 0 0 0.05

Hap_41 0 0 0 0 0 0 0.05

Hap_42 0 0 0 0 0 0 0.05

Hap_43 0 0 0 0 0 0 0.05

Hap_44 0 0 0 0 0 0 0.05

Hap_45 0 0 0 0 0 0 0.05
